# Loss of Function of mtHsp70 Chaperone Variants Leads to Mitochondrial Dysfunction in Congenital Sideroblastic Anemia

**DOI:** 10.3389/fcell.2022.847045

**Published:** 2022-02-16

**Authors:** Vinaya Vishwanathan, Patrick D’Silva

**Affiliations:** Department of Biochemistry, New Biological Sciences Building, Indian Institute of Science, Bangalore, India

**Keywords:** mortalin, molecular chaperone, mtHsp70, Ssc1, protein folding, congenital sideroblastic anemia

## Abstract

Congenital Sideroblastic Anemias (CSA) is a group of rare genetic disorders characterized by the abnormal accumulation of iron in erythrocyte precursors. A common hallmark underlying these pathological conditions is mitochondrial dysfunction due to altered protein homeostasis, heme biosynthesis, and oxidative phosphorylation. A clinical study on congenital sideroblastic anemia has identified mutations in mitochondrial Hsp70 (mtHsp70/Mortalin). Mitochondrial Hsp70 plays a critical role in maintaining mitochondrial function by regulating several pathways, including protein import and folding, and iron-sulfur cluster synthesis. Owing to the structural and functional homology between human and yeast mtHsp70, we have utilized the yeast system to delineate the role of mtHsp70 variants in the etiology of CSA’s. Analogous mutations in yeast mtHsp70 exhibited temperature-sensitive growth phenotypes under non-respiratory and respiratory conditions. *In vivo* analyses indicate a perturbation in mitochondrial mass and functionality accompanied by an alteration in the organelle network and cellular redox levels. Preliminary *in vitro* biochemical studies of mtHsp70 mutants suggest impaired import function, altered ATPase activity and substrate interaction. Together, our findings suggest the loss of chaperone activity to be a pivotal factor in the pathophysiology of congenital sideroblastic anemia.

## Introduction

Congenital Sideroblastic Anemia (CSA) is a heterogeneous group of inherited erythropoietic disorders and is characterized by the presence of abnormal erythrocyte precursors or ring-sideroblasts in the bone marrow. The cytological feature of sideroblasts is the pathological accumulation of iron-laden mitochondria around the nucleus giving the characteristic ring-like appearance (Abu-Zeinah and DeSancho, 2020). Within the cell, mitochondria house the molecular machinery that is required in the various stages of heme and Fe-S cluster synthesis, and thus, occupy a central role in iron metabolism (Ponka, 1999; Lill, 2009; Ye and Rouault, 2010). The first step in heme synthesis, occurs in the mitochondrial matrix. Furthermore, the Fe-S clusters assembled within the mitochondria serve as co-factors to several key proteins many of which are post-translational regulators of iron metabolism. Thus, it is implicit that any perturbation in mitochondrial function including impaired synthesis of heme, Fe-S cluster biogenesis, or impaired synthesis and/or import of mitochondrial proteins could contribute to the onset and progression of congenital sideroblastic anemia (Ducamp and Fleming, 2019; Fujiwara and Harigae, 2019). A genetic screen of patients with congenital sideroblastic anemia has revealed several mutations in the long arm of chromosome 5, specifically in the HSPA9 gene which codes for the mitochondrial Hsp70 protein (Schmitz-Abe et al., 2015). Most of the disease phenotypes were due to mild loss-of-function mutations. In severe cases, patients presented with a null allele in *trans* with a loss-of-function mutant. Furthermore, a non-coding region SNP (Single Nucleotide Polymorphism) in the gene which reduces mRNA expression was also found in the genetic analysis (Schmitz-Abe et al., 2015).

mtHsp70 is a ubiquitously expressed chaperone, highly conserved across species and plays a critical role in the biogenesis of mitochondria. It performs two key functions; import and folding of protein (Craig et al., 1989; Kang et al., 1990; Gambill et al., 1993; Liu et al., 2001). In addition, mtHsp70 actively engages in refolding misfolded proteins and preventing their aggregation, thereby maintaining protein quality control (Iosefson et al., 2012; Böttinger et al., 2015). mtHsp70 consists of two domains, the N-terminal Nucleotide Binding Domain (NBD) and C-terminal Substrate Binding Domain (SBD), connected by a flexible linker that is essential for inter-domain communication (Mayer and Bukau, 2005; Vogel et al., 2006). The cycling of substrate proteins between the ATP- and ADP-bound state of mtHsp70 aids in achieving the native folded conformation of the substrate. Further, mtHsp70s have co-evolved with several co-chaperone partners (J-proteins and Nucelotide Exchange Factor, NEF) to form specialized networks that carry out several functions within the mitochondria (Mayer and Bukau, 2005; Kampinga and Craig, 2010).

The human mitochondria have a single mtHsp70 (also referred to as mortalin/Grp75/PBP74) (Kaul et al., 2002; Wadhwa et al., 2002), while the yeast mitochondria possess three paralogs Ssc1, Ssq1, and Ecm10. Human mtHsp70 and yeast Ssc1 perform all the major housekeeping functions and share 66% identity and 82% similarity (Craig et al., 1989; Kang et al., 1990; Liu et al., 2001). In yeast, together with its co-chaperones, Ssc1 plays a pivotal role in maintaining protein quality control within the mitochondria by regulating the vectorial import of pre-proteins across the inner membrane and folding of proteins within the matrix (Laloraya et al., 1995; Westermann et al., 1996; Horst, 1997; D'Silva et al., 2003). While Ssq1 plays a critical role in Fe-S biogenesis, Ecm10 has been reported to be involved in mitochondrial DNA (mtDNA) maintenance and cell wall biogenesis (Baumann et al., 2000; Voisine et al., 2000; Dutkiewicz et al., 2003; Sakasegawa et al., 2003; Vickery and Cupp-Vickery, 2007). Interestingly, in humans, the single mtHsp70 in concert with multiple protein partners performs all the aforementioned functions (Kaul et al., 2002). Alterations in the function and levels of mtHsp70 in humans can affect multiple cellular processes. Mutations in mortalin are implicated in the onset and progression of neurodegenerative diseases including Alzheimer’s disease (AD) and Parkinson’s disease (PD). Reduced expression levels of mortalin leading to mitochondrial dysfunction is frequently observed in Parkinson’s disease (Jin et al., 2006; De Mena et al., 2009; Burbulla et al., 2010; Park et al., 2014; Wentink et al., 2019). Further, mortalin is also implicated in cancer progression and metastasis. Elevated levels of mortalin are reported in several cancers and are associated with poor prognosis and survival (Wadhwa et al., 2002; Dundas et al., 2005; Wadhwa et al., 2006; Yun et al., 2017). Therefore, it is plausible to speculate that the mutations in mtHsp70 could be a contributing cause in the pathophysiology of congenital sideroblastic anemia. However, the exact mechanisms of defects ensuing due to CSA-associated mtHsp70 variants remain to be explored.

Owing to the multiple functions of human mtHsp70, it is challenging to attribute the effects of the mutations to a specific function of mtHsp70. Given that yeast possesses three mtHsp70 paralogs, each involved in specialized functions, it would be possible to isolate the consequence of the mutations in a particular pathway. Yeast serves as an optimal model system due to the conservation of its cellular pathways with higher eukaryotes and the ease of genetically manipulating its genome. Thus, to specifically delineate the effects of CSA- associated mtHsp70 variants on the protein quality control and import, we have utilized the yeast model system and generated analogous mutations in Ssc1. Our findings indicate CSA mutations in Ssc1 lead to temperature-sensitive phenotype in non-respiratory and respiratory media. *In vivo* analyses indicate alterations in the mitochondrial content, functionality, and morphology, in the CSA mutants. Further, in the haploid state, these mutations exhibit compromised protein function, altered redox balance, and increased sensitivity to oxidative stress, resulting in the loss of protein quality control and mitochondrial dysfunction, and the subsequent manifestation of the pathologies associated with CSA.

## Materials and Methods

### Yeast Strains, Plasmids, and Genetic Analysis

For genetic and *in vivo* analyses, the yeast haploid strain DG252 (*trp1–1 ura3–1 leu2–3*, *112his3–11*, *15 ade2–1 can1–100 GAL2+ met2-Δ1 lys2-Δ2 ssc1ΔCla*I::LEU2) containing the plasmid *pRS316:SSC1* was used (Voisine et al., 1999). This strain was gifted by Prof. E A Craig, University of Wisconsin-Madison. The mutations in SSC1 were generated by PCR-based Quik-Change site-directed mutagenesis, using high-fidelity Pfu Turbo DNA Polymerase (Stratagene) (Tables 1, 2). All the site- specific mutations were confirmed by sequencing at AgriGenome labs, India. Mutants were transformed into the yeast strain, selected on minimal medium and subjected to counter selection on 5-fluoorotic acid medium (5-FOA) to ensure a single copy of SSC1 (cloned in a plasmid *pRS314:SSC1*). For heterologous protein expression and purification using *E. coli*, C-terminal His-tagged ORF (excluding the mitochondrial targeting sequence) of Ssc1 and its mutants was cloned into pRSFDuet-1 vector. To maintain protein solubility, Ssc1 was co-expressed with yeast Zim17 (Pareek et al., 2011) (Table 3).

**TABLE 1:** List of strains used in the study.

Name	Source	Genotype
*DG252*	Voisine et.al	*MATα trp1–1 ura3–1 leu2–3, 112 his3–11, 15 ade2–1 can1–100 GAL2*+ *met2-Δ1 lys2-Δ2 ssc1ΔCla*I::LEU2 pRS316-*SSC1*
WT *Ssc1*	Goswami et al.	*MATα trp1–1 ura3–1 leu2–3, 112 his3–11, 15 ade2–1 can1–100 GAL2*+ *met2-Δ1 lys2-Δ2 ssc1ΔCla*I::LEU2 pRS314-*SSC1*
^*ssc1*^S177L	This study	*MATα trp1–1 ura3–1 leu2–3*, *112 his3–11*, *15 ade2–1 can1–100 GAL2*+ *met2-Δ1 lys2-Δ2 ssc1ΔCla*I::LEU2 pRS314-*ssc1*_*S177L*_
^*ssc1*^V189P	This study	*MATα trp1–1 ura3–1 leu2–3*, *112 his3–11*, *15 ade2–1 can1–100 GAL2*+ *met2-Δ1 lys2-Δ2 ssc1ΔCla*I::LEU2 pRS314-*ssc1*_*V189P*_
^*ssc1*^G365S	This study	*MATα trp1–1 ura3–1 leu2–3*, *112 his3–11*, *15 ade2–1 can1–100 GAL2*+ *met2-Δ1 lys2-Δ2 ssc1ΔCla*I::LEU2 pRS314-*ssc1*_*G365S*_
^*ssc1*^E392K	This study	*MATα trp1–1 ura3–1 leu2–3*, *112 his3–11*, *15 ade2–1 can1–100 GAL2*+ *met2-Δ1 lys2-Δ2 ssc1ΔCla*I::LEU2 pRS314-*ssc1*_*E392K*_
^*ssc1*^T516K	This study	*MATα trp1–1 ura3–1 leu2–3*, *112 his3–11*, *15 ade2–1 can1–100 GAL2*+ *met2-Δ1 lys2-Δ2 ssc1ΔCla*I::LEU2 pRS314-*ssc1*_*T516K*_
^*ssc1*^Q554K	This study	*MATα trp1–1 ura3–1 leu2–3*, *112 his3–11*, *15 ade2–1 can1–100 GAL2*+ *met2-Δ1 lys2-Δ2 ssc1ΔCla*I::LEU2 pRS314-*ssc1*_*Q554K*_

**TABLE 2:** List of primers used in the study.

Name (primer)	Sequence (5′-3′)
ssc1 S177L SDM—Forward	GCT​TAT​TTC​AAC​GAC​TTG​CAA​AGA​CAA​GCT​ACT​AAA​GAC
ssc1 S177L SDM—Reverse	GTC​TTT​AGT​AGC​TTG​TCT​TTG​CAA​GTC​GTT​GAA​ATA​AGC
ssc1 V189P SDM—Forward	CGC​AGG​CCA​AAT​TCC​TGG​TTT​GAA​CGT​TTT​AC
ssc1 V189P SDM—Reverse	GTA​AAA​CGT​TCA​AAC​CAG​GAA​TTT​GGC​CTG​CG
ssc1 G365S SDM—Forward	CTT​ATT​GGT​CGG​TTC​TAT​GTC​CAG​AAT​GCC
ssc1 G365S SDM—Reverse	GGC​ATT​CTG​GAC​ATA​GAA​CCG​ACC​AAT​AAG
ssc1 E392KSDM—Forward	CGT​CAA​CCC​AGA​TAA​AGC​TGT​TGC​CAT​TGG
ssc1 E392K SDM—Reverse	CCA​ATG​GCA​ACA​GCT​TTA​TCT​GGG​TTG​ACG
ssc1 T516K SDM—Forward	GCT​AGA​GAC​AAA​GCT​AAA​AAC​AAA​GAT​TC
ssc1 T516K SDM—Reverse	GAA​TCT​TTG​TTT​TTA​GCT​TTG​TCT​CTA​GC
ssc1 Q554K SDM - Forward	GAA​GCT​AGA​AAA​AAA​GCC​ATC​GAA​ACT​GCC
ssc1 Q554K SDM—Reverse	GGC​AGT​TTC​GAT​GGC​TTT​TTT​TCT​AGC​TTC

**TABLE 3:** List of plasmid constructs used in the study.

Name	Vector	Restriction sites	Source	Description
*SSC1* WT	pRS314	PstI/BamHI	Craig Lab	WT *SSC1* cloned under its native promoter
S177L	pRS314	PstI/BamHI	This study	S177L mutant cloned under its native promoter
V189P	pRS314	PstI/BamHI	This study	V189P mutant cloned under its native promoter
G365S	pRS314	PstI/BamHI	This study	G365S mutant cloned under its native promoter
E392K	pRS314	PstI/BamHI	This study	E392K mutant cloned under its native promoter
T516K	pRS314	PstI/BamHI	This study	T516K mutant cloned under its native promoter
Q554K	pRS314	PstI/BamHI	This study	Q554Kmutant cloned under its native promoter
*SSC1* WT	pRSFDuet-1	NdeI/XhoI	This study	His-tagged WT Ssc1 protein co-expressed with Zim17
S177L	pRSFDuet-1	NdeI/XhoI	This study	His-tagged S177L mutant protein co-expressed with Zim17
V189P	pRSFDuet-1	NdeI/XhoI	This study	His-tagged V189P mutant protein co-expressed with Zim17
G365S	pRSFDuet-1	NdeI/XhoI	This study	His-tagged G365S mutant protein co-expressed with Zim17
E392K	pRSFDuet-1	NdeI/XhoI	This study	His-tagged E392K mutant protein co-expressed with Zim17
T516K	pRSFDuet-1	NdeI/XhoI	This study	His-tagged T516K mutant protein co-expressed with Zim17
Q554K	pRSFDuet-1	NdeI/XhoI	This study	His-tagged Q554K mutant protein co-expressed with Zim17
Mdj1	pRSFDuet-1	NdeI/XhoI	D’Silva lab	His-tagged Mdj1 protein

### Flow Cytometry and Analysis

The total and functional mitochondrial mass were determined using 10 µM NAO 10-Nonyl Acridine Orange (NAO, Molecular Probes) and 8.75 µM Tetramethylrhodamine, ethyl ester (TMRE, Molecular probes) respectively. To measure the cellular and mitochondrial ROS, cells were stained with 100 µM DCFDA (Calbiochem) and 5 µM MitoSOX (Molecular probes) respectively. Staining was carried out at 30°C (24°C for G365S and E392K) for 20 min, followed by FACS analysis. Fluorescence intensities of 10,000 cells were acquired and analyzed using WinMDI 2.9 software.

### Visualization of Mitochondrial Morphology

For mitochondrial morphology study, cells were transformed with an RFP fusion protein (MTS-mCherry), containing an N-terminal mitochondrial targeting sequence that localizes to the mitochondria (Bankapalli et al., 2015). Cells expressing MTS-mCherry were grown up to the mid-log phase and subjected to microscopic analysis using the Delta vision Elite imaging system (GE Healthcare). Images were processed using software 6.1.3 software (GE Healthcare).

### Statistical Analysis

Error bars in the bar charts represent the standard error of mean (Mean ± S.D). Significance testing (in comparison with the wild-type) was performed using One-way ANOVA with Dunnet’s Multiple Comparison Post Test. Asterisks used in the figures to denote the significance represent the following: *p ≤ 0.05; **p ≤ 0.01; ***p ≤ 0.001.

### Miscellaneous

Mitochondria isolation and the purification of proteins Ssc1 and Mdj1 was done using standard protocols (Sinha et al., 2010; Goswami et al., 2012). *In vivo* precursor accumulation assay (Kang et al., 1990), *in vitro* single-turnover ATPase activity (Goswami et al., 2010), and peptide-binding assays (Azem et al., 1997; Liu et al., 2001) were performed as previously described. Antibodies Hsp60 (1:5,000 dilution) and Ydj1 (1:8,000 dilution) were kind gifts from Prof. E.A. Craig’s laboratory (University of Wisconsin–Madison, Madison, WI). Antibodies against Ssc1 (1:5,000 dilution) and Tim23 (1:5,000 dilution) were raised against pure proteins in rabbits (Sinha et al., 2010) (Imgenex, India). Immunoblot assays were performed using ECL reagents (Bio-Rad) according to the manufacturer’s instructions.

## Results

### Analogous Congenital Sideroblastic Anemias Mutations, G365S and E392K, in Yeast mtHsp70 Result in Compromised Growth

**FIGURE 1:**
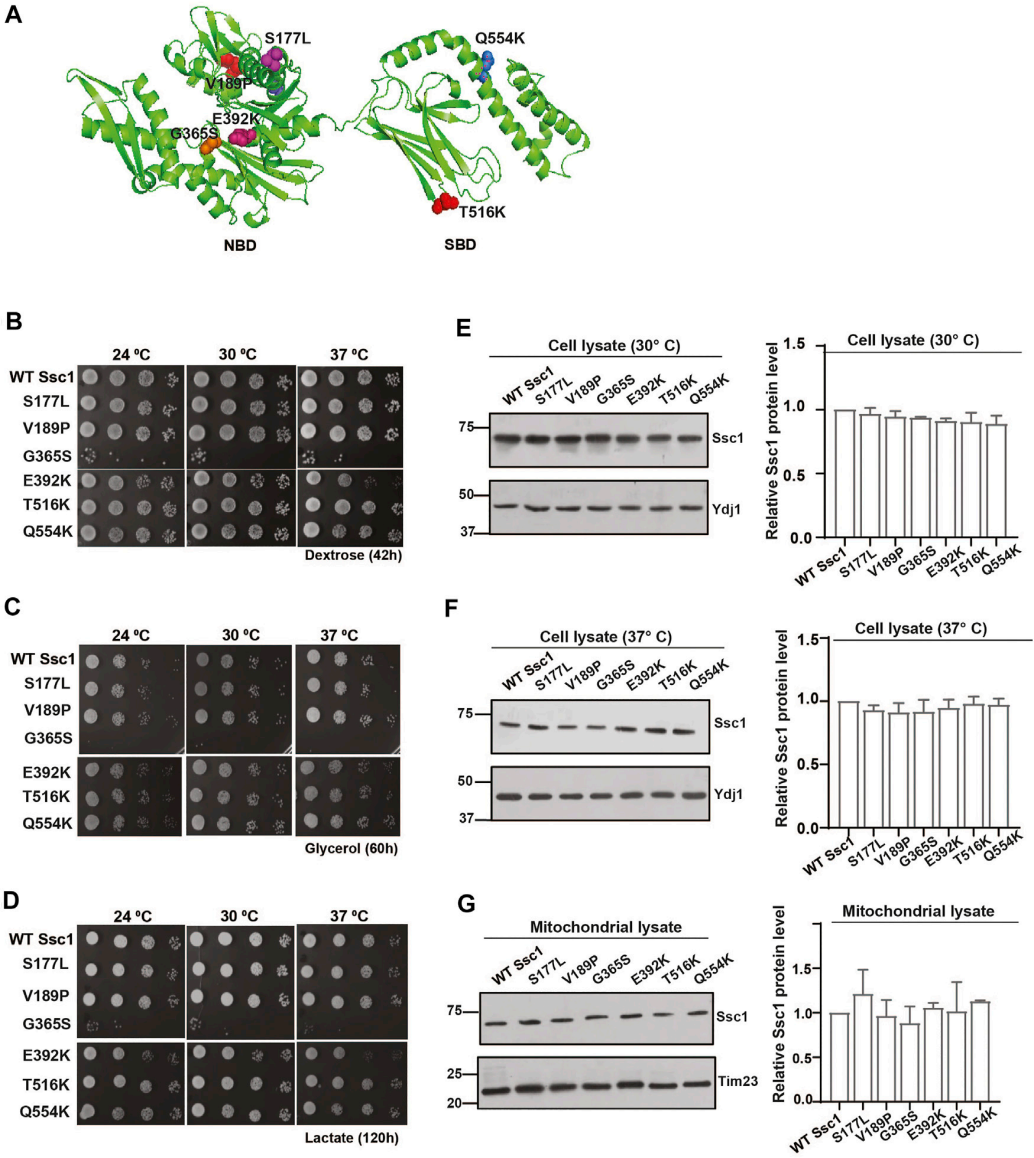
Growth phenotype and protein levels of CSA variants in yeast. **(A)** The residues analogous to the variants in congenital sideroblastic anemia are labeled and represented as colored spheres. The modelled structure of full-length yeast Ssc1 is based on the bacterial DnaK structure (PDB ID: 2KHO). **(B–D)** Analysis of growth. Equivalent cells diluted ten-fold were spotted on rich media containing **(B)** dextrose **(C)** glycerol and **(D)** lactose and incubated at the indicated temperatures. **(E–G)** Steady levels of Ssc1 mutants. Cell lysates of equivalent cells grown at **(E)** permissive temperature and **(F)** non permissive temperature (37°C) were resolved by SDS-PAGE and the expression levels of Ssc1 were determined by immunoblotting using anti-Ssc1 antibodies. Cytosolic protein Ydj1 was used as the loading control. **(G)** Levels of mutants targeted to the mitochondria was measured by immunoblot analysis of 50 µg of isolated mitochondria. Tim23 was used as the mitochondrial loading control. The levels of Ssc1 were quantified using Multigauge software, normalized with respect to the wild-type and represented as Mean ± S.D of three independent experiments.

The mtHsp70 variants observed in the clinical screen of Congenital Sideroblastic Anemia are mapped to residues in both the NBD (S177L, V189P, G365S, and E392K) and the SBD (T516K and Q554K) (Figure 1A). To dissect the effects of these mutations on the mitochondrial protein quality control, we generated analogous mutations in the corresponding residues of Ssc1, an ortholog of human mtHsp70. To analyze the growth phenotype, serial dilutions of strains expressing either the wild-type or mutant were spotted on non-respiratory medium (YPD) and respiratory media (YPG and YPL) and incubated at various temperatures. Upon spot analysis, as compared to the wild-type, the G365S mutant exhibited sensitivity at all temperatures (consistent with the previous report) (Schmitz-Abe et al., 2015), whereas the E392K mutant showed reduced growth at 34°C and 37°C on YPD medium (Figure 1B). Phenotype analysis under respiratory conditions showed a reduction in the growth of G365S (all temperatures) and E392K (non-permissive temperatures) mutants as compared to the wild-type strain (Figures 1C,D). Interestingly, the other Ssc1 mutants showed growth comparable to that of wild-type Ssc1 at all temperatures irrespective of the media. Further to ascertain if the observed growth defect was due to an alteration in the protein expression levels, we evaluated the levels of Ssc1 in the whole cell lysates at both permissive (Figure 1E) and non-permissive temperatures (Figure 1F). Immunoblot analysis revealed that the difference in the growth of the mutants was not due to changes in protein expression levels. Further analysis of mitochondrial lysates revealed that equivalent levels of the Ssc1 mutants were localized to the mitochondria (Figure 1G) suggesting that the observed growth phenotype is not due to the difference in the expression or localization of the mtHsp70 variants but could be due to an alteration in the protein function.

### Congenital Sideroblastic Anemias Variants Exhibit Alteration in Import Function, ATPase Activity and Substrate Interaction

**FIGURE 2:**
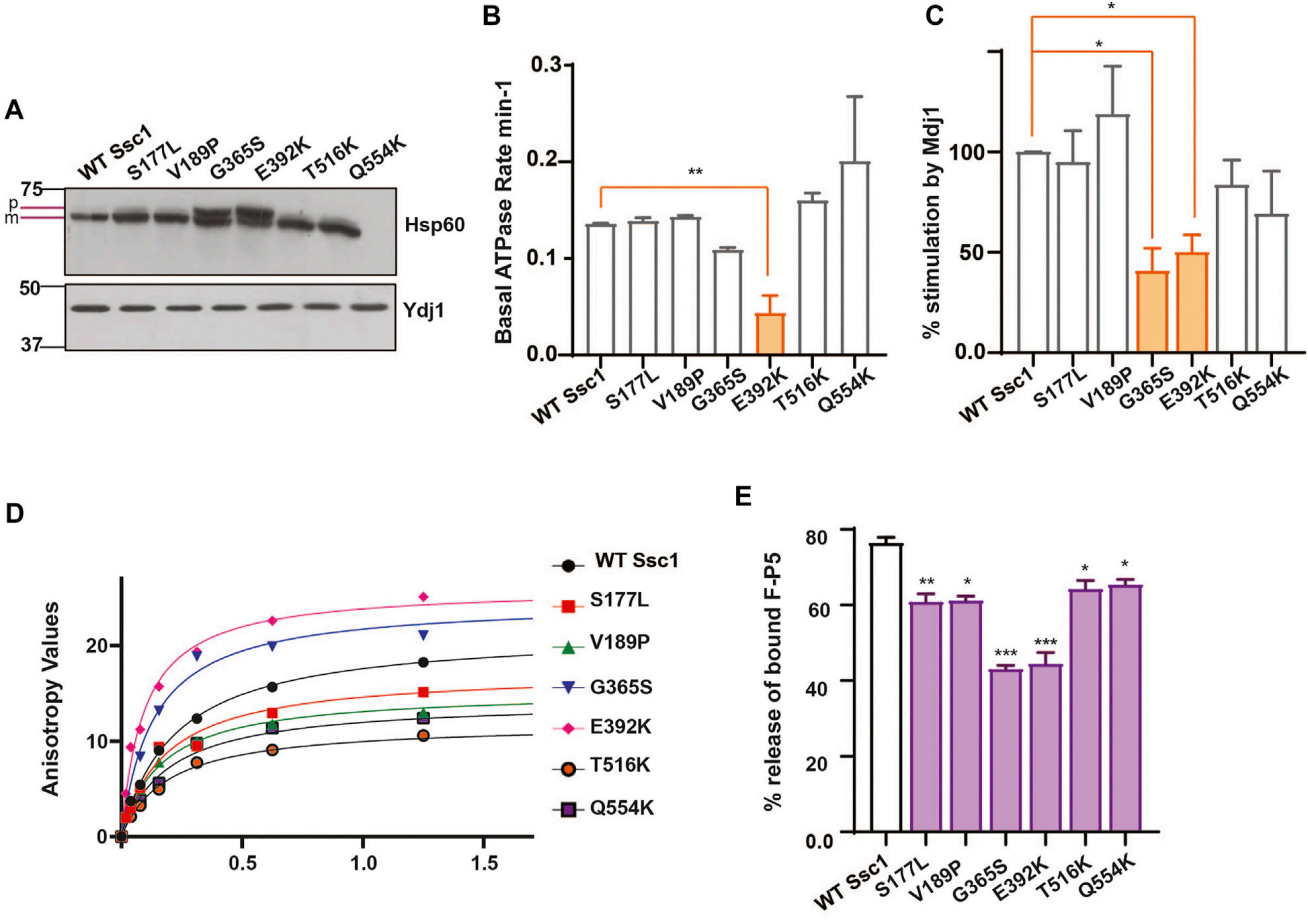
Ssc1 mutants exhibit loss of function **(A)** *In vivo* precursor accumulation assay. Cells were grown at their respective permissive temperatures up to early log phase and shifted to 37°C for 4 h. The cell lysates were separated by SDS-PAGE and analyzed by immunoblotting using anti-Hsp60 antibodies to determine the precursor (p) and mature (m) forms. Cytosolic protein Ydj1 was used as the loading control. **(B,C)** Measurement of ATPase activity.1 µM of preformed Ssc1-radiolabeled ATP was incubated with **(B)** buffer alone and **(C)** 2 µM of purified Mdj1. The rate of ATP hydrolysis was calculated at indicated time points and fitted to a one-phase exponential equation **(C)** The fold stimulation of ATPase activity was normalized with respect to the basal rates. **(D,E)** Substrate interaction studies. **(D)** 25 nM of fluorescein labeled peptide containing CALLLSAPRR sequence (F-P5) was incubated with increasing concentrations of purified Ssc1 proteins and allowed to attain binding equilibrium. Post equilibrium, anisotropy values for every protein concentration was measured and fitted to one-site binding equation to obtain the dissociation constant *K*_d_. **(B)** 1000- fold excess of ATP was added to the Ssc1-FP5 complex post binding equilibrium. The decrease in anisotropy was monitored and the maximal decrease was noted to calculate the percentage of peptide (F-P5) released upon ATP binding. Data plotted as Mean ± S.D of two independent experiments. Curve fitting analyses were done using Graph Pad Prism.

To assess the import function of Ssc1, we performed *in vivo* precursor accumulation assay wherein any compromise in the Ssc1 function would lead to the accumulation of unprocessed precursors of nuclear-encoded proteins. Briefly, cells expressing Ssc1 mutants were grown at permissive temperatures and subjected to a heat shock at 37°C for 4 h. Immunoblot analyses were performed using anti-Hsp60 antibody to determine the accumulation of unprocessed forms of Hsp60. Hsp60 was chosen as a probe due to its abundant expression at permissive and non-permissive temperatures. Interestingly, *in vivo* import assay revealed accumulation of Hsp60 precursors in the G365S and E392K mutants (Figure 2A), suggesting a strong defect in the import function, which aligns well with their observed growth phenotype.

mtHsp70 chaperone activity is driven by iterative substrate binding coupled to ATP binding and hydrolysis. Therefore, we performed *in vitro* assays to determine the effect of Ssc1 mutations on its ATPase activity and/or substrate binding property. To evaluate the ATPase activity, purified proteins were subjected to single-turnover ATPase assay to determine the rate of ATP hydrolysis. Intriguingly, while most of the mutants did not show any significant alterations, the E392K (∼50%) mutant showed significant reduction in the basal ATP hydrolysis rates (Figure 2B). A similar reduction in the ATPase activity was observed for the G365S and E392K mutants in the presence of J-protein, Mdj1 (Figure 2C). Further to understand how the mutations affect the substrate binding properties of Ssc1, we utilized a fluorescence-based assay using a fluorescein labelled peptide, F-P5. Fluorescence analysis indicated the wild-type to have a *K*_**d**_ value of 0.2160 ± 0.001 which is in agreement with the earlier report (Liu et al., 2001). In comparison to the wild-type, the G365S and E392K mutants showed a ∼1.6-fold and ∼2.2-fold increased affinity for the substrate (Figure 2D; Table 4). Further, the addition of ATP to the Ssc1-FP5 complex revealed a decrease in the ATP-mediated substrate release for all the mutants, in particular the G365S and E392K mutants showed a significant reduction (∼40% peptide release) (Figure 2E). Together these results suggest that the CSA-associated mutations in mtHsp70 lead a loss of protein function.

**TABLE 4:** List of the equilibrium dissociation constants (*K*_d_) for F-P5 for yeast Ssc1 CSA-variants proteins.

Protein	*K*_d_ (µM) ± SD
*SSC1* WT	0.2160 ± 0.001
S177L	0.1840 ± 0.001
V189P	0.1857 ± 0.02
G365S	0.1312 ± 0.002
E392K	0.0942 ± 0.001
T516K	0.1958 ± 0.004
Q554K	0.1904 ± 0.002

### Congenital Sideroblastic Anemias Associated Mutations Perturb Mitochondrial Network, Content, and Functionality

**FIGURE 3:**
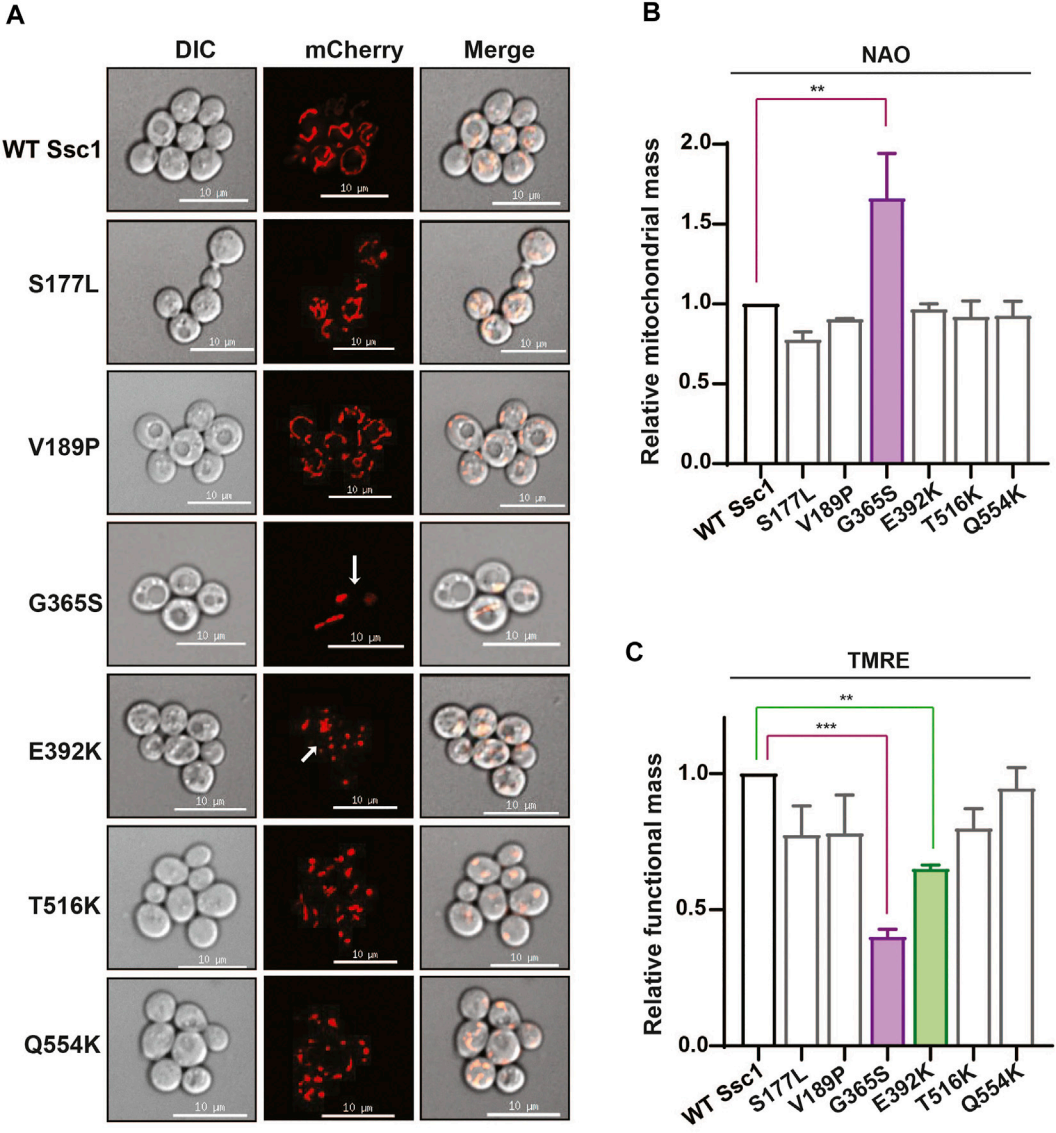
CSA mutations perturb mitochondrial physiology. **(A)** Mitochondrial morphology. Wild-type and mutant cells were transformed with RFP fusion protein, MTS-mCherry to visualize the mitochondrial network. Images were captured at identical exposures and analyzed using SoftWoRx 6.1.3 software. Scale 10 µm. **(B,C)** Quantification of total and functional mitochondrial mass. Equivalent cells grown up to mid log phase were stained with **(B)** 10 µm NAO and **(C)** 8.75 µM TRME to quantify the total and functional mitochondrial mass respectively. Fluorescence median intensities of 10,000 cells were obtained, normalized with respect to the wild-type represented as Mean ± S.D of three independent experiments.

To understand how mutations in mtHsp70 affect mitochondrial physiology, we evaluated multiple parameters that are indicative of the overall organelle health. Foremost, we visually analyzed the mitochondrial morphology using an RFP-fusion protein, MTS-mCherry (Figure 3A). Microscopy analyses revealed all the mutants, except for G365S and E392K exhibited a reticular/intermediate mitochondrial network similar to that observed in the wild-type. However, the G365S and E392K mutants displayed a punctate mitochondrial network. Subsequently, we measured the mitochondrial content by staining the cells with a cardiolipin-specific dye, 10-*N*-Nonylacridine orange (NAO) followed by flow cytometry. Analysis of the fluorescence intensity values indicated an approximately 1.5-fold increase in total mitochondrial content in the G365S mutant while the other mutants did not show any significant alteration in the mitochondrial content as compared to the wild-type (Figure 3B). Further, we assessed the functionality of mitochondria by staining the cells with Tetramethylrhodamine, ethyl ester (TMRE). Uptake of the TMRE dye depends on the mitochondrial inner membrane potential (Δψ) and thus is indicative of mitochondrial functionality. Staining with TMRE revealed ∼50% reduction of the functional mitochondrial in G365S mutant. Interestingly, the E392K mutant also exhibited approximately 25% reduction in the functional mitochondria (Figure 3C). Several studies have established mitochondrial puncta/fission to be observed upon the loss of mitochondrial membrane potential. Thus, these findings corroborate the punctate network observed in the G365S and E392K mutants and indicate that CSA-associated mutations in Ssc1 impair mitochondrial network, content, and functionality.

### Ssc1 Mutants Increase Cellular ROS Levels and Oxidative Stress Sensitivity

**FIGURE 4:**
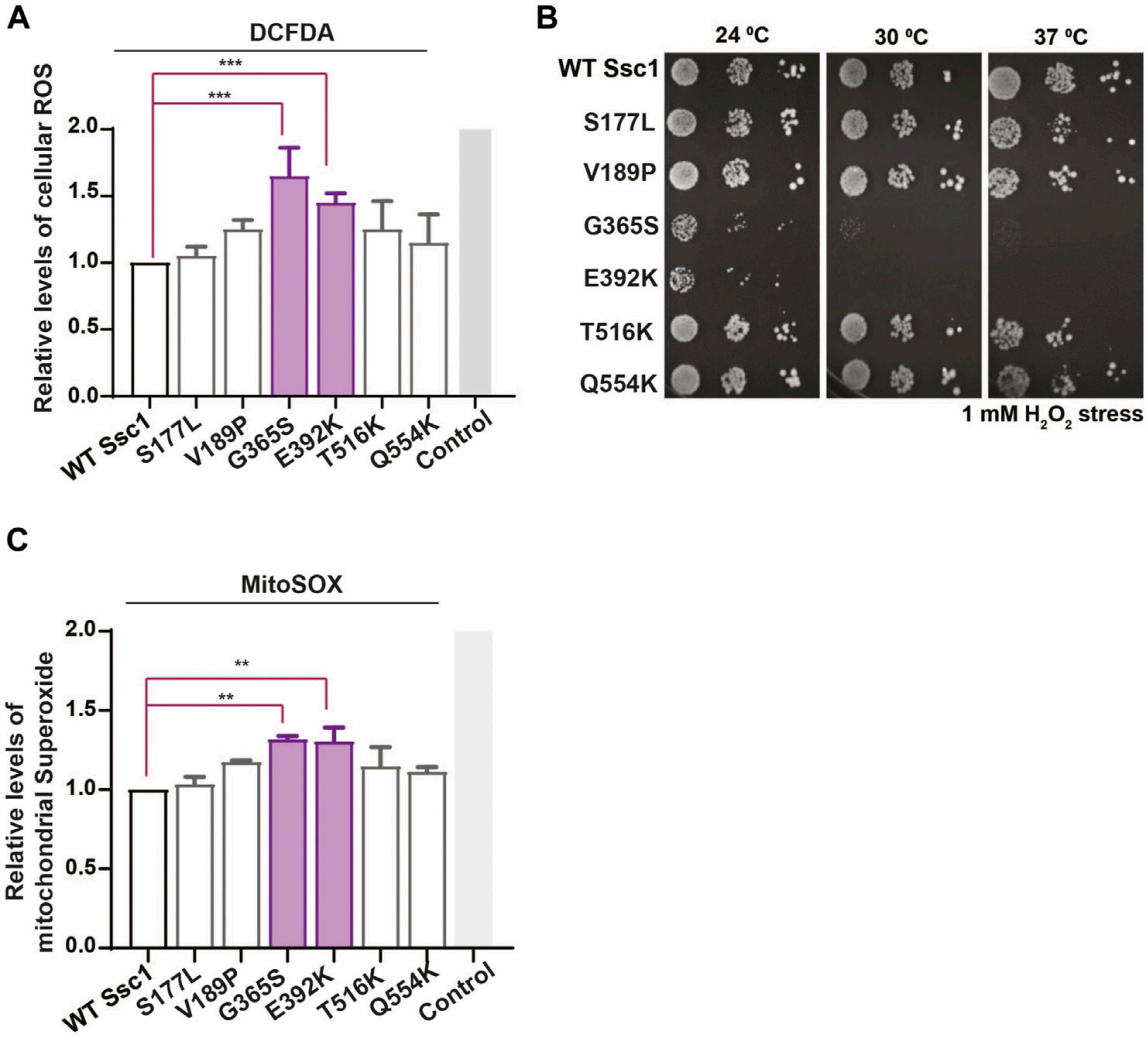
Mutations in Ssc1 alter the redox balance and increase oxidative sensitivity. **(A)** Analysis of cellular ROS. Equivalent cells were stained with 100 µm H_2_DCFDA to quantify the total cellular ROS. Fluorescence median intensities of 10,000 cells were obtained, normalized with respect to the wild-type and represented as Mean ± S.D of two independent experiments. Wild-type cells treated with 1 mM H_2_O_2_ served as a control **(B)** Sensitivity to oxidative stress. Mid log phase cells were treated with 1 mM H_2_O_2_ for 1 h at the respective permissive temperature, spotted on YPD medium and incubated at the indicated temperatures for 96 h. Sensitivity to oxidative stress was scored based on growth at various temperatures. **(C)** Mitochondrial superoxide levels were quantified by staining equal number of cells with 5 µM MitoSox. Wild-type cells treated with 100 µM Antimycin A served as a control. Fluorescence median intensities of 10,000 cells were obtained, normalized with respect to the wild-type and represented as Mean ± S.D of two independent experiments.

Altered mitochondrial functionality has been associated with a perturbation in the cellular redox levels. Thus, to evaluate whether the CSA-associated mutants show any alterations in the oxidative balance, we stained the yeast cells with H_2_DCFDA dye. H_2_DCFDA is a cell-permeable dye that fluoresces upon being cleaved by the cellular esterases and subsequently oxidized by peroxide ions. Interestingly, DCFDA staining revealed a significant increase in the intrinsic ROS levels in the G365S and E392K mutants (Figure 4A). Additionally, to determine the sensitivity of the Ssc1 mutants to extraneous oxidative stress, cells were treated with 1 mM H_2_O_2_ for 1 h at 30°C (24°C for G365S and E392K) prior to spotting on YPD medium (Goswami et al., 2012; Bankapalli et al., 2015). The G365S and E392K mutants displayed a severely compromised growth upon being subjected to external oxidative stress (Figure 4B). This is in corroboration with the FACS analyses wherein the Ssc1 mutants exhibited increased cellular ROS levels.

Further to analyze the mitochondrial superoxide levels, the cells were stained with MitoSox and analyzed by FACS. MitoSox rapidly accumulates within the mitochondria and fluoresces upon oxidation by superoxide ions. FACS analyses indicate an increase in the mitochondrial superoxide levels in the G365S and E392K mutants (Figure 4C). Together these results indicate CSA-associated mutants alter the function of mthsp70 resulting in mitochondrial dysfunction and redox imbalance, which contribute to the onset and progression of congenital sideroblastic anemia.

## Discussion

The onset and progression of congenital sideroblastic anemia have been strongly associated with mitochondrial dysfunction. CSA associated mutations in mtHsp70 map to both the Nucleotide Binding Domain (NBD) and Substrate Binding Domain (SBD), some of which are in conserved regions of the protein (Schmitz-Abe et al., 2015). Utilizing the yeast system, we have generated analogous mutations in Ssc1 to evaluate how CSA variants affect the general protein homeostasis within the mitochondria, Also, the location of most of the variants in conserved regions of the protein indicates that the possible defects associated with the mtHsp70 mutants would affect the chaperone function similarly across species.

Analogous mutations in Ssc1 revealed two mutants to result in a conditional phenotype with compromised growth at non-permissive temperatures. Furthermore, cells expressing these mutations showed multiple phenotypes including 1) defects in preprotein import across the inner mitochondrial membrane 2) alteration in mitochondrial morphology 3) reduction in functional mitochondrial mass 4) enhanced intrinsic ROS levels, and 5) increased susceptibility to oxidative stress. Further, the cellular levels of Ssc1 mutants were comparable to that of wild type suggesting that the defects arising due to the mutations are at functional level. *In vivo* analysis for efficient preprotein import reveals two variants G365S and E392K to show precursor accumulation indicating a defective import function. Furthermore*, in vitro* analyses indicate that the release of substrates from the SBD upon ATP binding was altered in all the mutants, in particular the G365S and E392K mutants. Interestingly, both the variants also exhibited significant reduction in their basal ATP hydrolysis rate and stimulation by J-protein. At the protein level, the Gly365 and Glu392 residues are located at conserved regions in the NBD that are critical for the interaction with ATP and ATPase activity respectively (Qi et al., 2013; Schmitz-Abe et al., 2015). Therefore, it is plausible that the mutations in these residues could result in the significant reduction of the ATPase activity and ATP-dependent release.

An impaired iron metabolism resulting in the accumulation of mitochondrial free iron is a hallmark feature of CSA. The redox active free iron catalyzes free radical formation via the Fenton chemistry resulting in elevated ROS levels and mitochondrial dysfunction (Thomas et al., 2009). Interestingly, our study indicates increased intrinsic cellular ROS levels and oxidative-stress sensitivity in the CSA mutants. Further, most of the proteins involved in heme metabolism and Fe-S cluster synthesis are nuclear-encoded and thus require mtHsp70 for their import and folding within the mitochondria. Based on the observed increase in the ROS levels and the loss of protein function, we speculate that the mutations in Ssc1 could perturb the mitochondrial iron metabolism. In case of the variants not exhibiting a significant alteration in growth could be due to the presence of the additional paralogs in the yeast mitochondria. However, unlike yeast, the human mitochondria possess a single mtHsp70 performing all the housekeeping functions and handling a proteome twice as that of lower eukaryotes. In line with this, mutations in human mtHsp70 can severely impair multiple mitochondrial processes including the heme metabolism and Fe-S cluster biogenesis and result in organelle dysfunction. Therefore, we hypothesize that the dysregulation of protein quality control due to altered chaperone function leads to dysregulated mitochondrial function and thereby impairs the iron metabolism resulting in the disease phenotypes observed in CSA.

In conclusion, our findings delineate the probable mechanism by which mutations in mtHsp70 contribute to the pathological symptoms and mitochondrial features observed in CSA. Furthermore, our study in the yeast model system specifically dissects the effects of human mtHsp70-variants on the overall mitochondrial protein quality control and homeostasis. These results can provide insights into better understanding the molecular mechanisms involved in the pathophysiology and progression of congenital sideroblastic anemia.

## Data Availability

The original contributions presented in the study are included in the article/supplementary material, further inquiries can be directed to the corresponding author.
